# Expression of thioredoxin 1 and peroxiredoxins in squamous cervical carcinoma and its predictive role in NACT

**DOI:** 10.1186/s12885-019-6046-x

**Published:** 2019-08-30

**Authors:** Haiyan Zhu, Xuejiao Tao, Lulu Zhou, Bo Sheng, Xuejie Zhu, Xueqiong Zhu

**Affiliations:** 1 Department of Obstetrics and Gynecology, the Second Affiliated Hospital of Wenzhou Medical University, No. 109 Xueyuan Xi Road, Wenzhou, 325027 Zhejiang China; 2 Department of Gynecology, the First Affiliated Hospital of Wenzhou Medical University, Shangcaicun Road, Wenzhou, 325000 China

**Keywords:** Thioredoxin 1, Peroxiredoxin 1, Peroxiredoxin 2, Neoadjuvant chemotherapy, Cervical squamous cancer

## Abstract

**Background:**

This study aims to investigate the expression of thioredoxin 1, peroxiredoxin 1 and peroxiredoxin 2 in bulky cervical squamous carcinoma and its predictive role in cisplatin-based neoadjuvant chemotherapy.

**Methods:**

Initially, the expression of thioredoxin 1, peroxiredoxin 1 and peroxiredoxin 2 protein was analyzed in 13 human cervical squamous cancer tissues and their paired adjacent non-cancerous tissues by western-blotting and immunohistochemistry. Then, correlation between the expression of thioredoxin 1, peroxiredoxin 1, peroxiredoxin 2 and responses to cisplatin-based neoadjuvant chemotherapy was analyzed in 35 paired tumor samples (pre- and post-chemotherapy) from bulky cervical squamous cancer patients by immunohistochemistry.

**Results:**

A clinical response occurred in 48.6% (17/35) of patients, including 14.3% (5/35) with a complete response and 34.3% (12/35) with a partial response. The expression of thioredoxin 1, peroxiredoxin 1 and peroxiredoxin 2 was much higher in cervical squamous cancer tissues compared with paired adjacent non-cancerous tissues by western-blotting and immunohistochemistry. Additionally, the expression of thioredoxin 1, peroxiredoxin 1 and peroxiredoxin 2 was significantly up-regulated in post-chemotherapy tissues compared to pre-chemotherapy cervical cancer tissues. High levels of thioredoxin 1, peroxiredoxin 1 and peroxiredoxin 2 were associated with a poor chemotherapy response in cervical squamous cancer patients.

**Conclusions:**

Thioredoxin 1, peroxiredoxin 1 and peroxiredoxin 2 are frequently over-expressed in cervical squamous cancer. High expression levels of these proteins were related to a poor response to cisplatin-based neoadjuvant chemotherapy. The present study is the first report that thioredoxin peroxidase system may serve as a prediction of the responses to neoadjuvant chemotherapy in cervical squamous cancer.

## Highlights

1. Thioredoxin 1, peroxiredoxin 1/ 2 are over-expressed in cervical squamous cancer.
2. High levels of thioredoxin 1 and peroxiredoxin 1/2 related to a poor chemotherapy response in cervical squamous cancer patients.
3. Thioredoxin peroxidase system may serve as a prediction of the responses to neoadjuvant chemotherapy in cervical squamous cancer.

## Background

The widespread uptake of screening for the prevention and early detection of cervical cancer has been largely successful in more developed countries. However, cervical cancer remains the second most commonly diagnosed cancer and third leading cause of cancer death among females in less developed countries, where a significant proportion of patients are diagnosed with locally advanced cervical cancer, and often have a poor prognosis [1]. Neoadjuvant chemotherapy (NACT) prior to surgery has been widely used for bulky or locally advanced cervical cancer. In a large majority of these patients, treatment is effective, which can shrink the tumor size, suppress micrometastases, and promote the safety and integrity of surgery [2]. In 20–25% of cases, however, this neoadjuvant chemotherapy fails to achieve tumor regression [2], furthermore, the prognosis of NACT-refractory patients would become worse due to the delay in curative treatment [3]. Therefore, there is an obvious need to identify and develop novel biomarkers which may aid in predicting chemotherapy responses, and thus to assess the treatment options of individual patients.

Recently, appreciable evidence suggested that some approaches of chemotherapy implicate the stimulation of intracellular reactive oxygen species (ROS) production to eliminate cancer cells [4–6], and thus ROS associated oxidative stress have been considered involvement in the development of chemoresistance [7]. Correspondently, human body produces several anti-oxidants that protect against ROS. Indeed, an increasing body of data suggests thioredoxin 1 (Trx 1) together with peroxiredoxins (Prxs), form the thioredoxin peroxidase system that scavenge peroxides, provide protective effects against ROS-induced damages, thereby reducing the efficacy of the treatment [8, 9].

Trx 1 is a 12-kDa protein with redox-active dithiol in the active site -Cys-Gly-Pro-Cys- that is ubiquitously present in the human body [10]. It is a defensive protein induced by various stresses and has anti-oxidative, anti-apoptotic and anti-inflammatory effects [9]. Trx 1 plays crucial roles in pathophysiological mechanisms of cancer and aberrant expression of Trx 1 has been detected in many forms of cancers [11], correlating with cancer development, metastasis, progression, survival and resistance to chemotherapy [10]. Trx 1 has been reported involved in paclitaxel -induced drug resistance in ovarian cancer A2780 cells [12] and cisplatin-induced resistance in pancreatic cancer cells [13]. Over-expressed human Trx was responsible for the development of cellular resistance to cispaltin in Jurkat T cells [14]. Until now, no study has evaluated the potential role of Trx 1 in cervical carcinogenesis as well as its implication in resistance to chemotherapy among squamous cervical cancer patients.

Trx 1 exerts its main function as an antioxidant mainly via its interactions with downstream peroxiredoxins [15]. Peroxiredoxins are a ubiquitous family of thiol-dependent peroxidases that catalyse the reduction of H_2_O_2_, and different alkyl hydroperoxides [16]. They are involved in a variety of important cellular functions and up-regulated in many human cancer types, contributing to the development of tumor progression and chemo-resistance [17]. However, whether peroxiredoxins expression correlates with chemoresistance in squamous cervical cancer patients is currently unknown.

In line with the above evidence, we hypothesis that chemotherapeutics can cause ROS, possibly resulting into the increase of antioxidant factors such as Trx1, Prx1 and Prx2, providing protective effects against ROS-induced damages, and therefore contribute to resistance development. In fact, our previous study has detected up-regulation of Prx 1 in squamous cervical cancer tissues after NACT relative to the level before chemotherapy by using proteomics profiling [18]. The aim of the current study is to determine the expression of Trx 1, Prx 1 and Prx 2 in squamous cervical carcinoma and their potential roles in predicting chemoresistance among stage IB2 or IIA2 squamous cervical cancer patients treated with neoadjuvant chemotherapy.

## Methods

### Patients and tissue specimens

All tissue samples were collected from the Second Affiliated Hospital of Wenzhou Medical University after informed written patient consent. This study was approved by the ethical committee of the Second Affiliated Hospital of Wenzhou Medical University and conducted according to the Helsinki declaration. At first, a total number of 13 squamous cervical cancer tissues and adjacent normal tissues were collected between January 2014 and April 2015 for the expression analysis of Trx 1, Prx 1 and Prx 2 by western-blotting and immunohistochemistry. The median age of the 13 patients is 49y (range, 36~74y). These patients are in stage IB1 (10 of 13 patients, 76.9%) and IIA1 (3 of 13 patients, 23.1%) with the median diameter of 2.13 ± 0.12. Ten cases were grade I, 2 cases were grade II and 1 case was grade III. Stage and grading were according to the International Federation of Gynecology and Obstetrics (FIGO) system. Paired tumor cervical cancer tissues and adjacent normal tissues from each patient were obtained after radical hysterectomy.

Another cohort (cohort 2) consisted of 35 patients with IB2 or IIA2 (bulky, primary tumor > 4 cm in diameter) squamous cervical cancer treated with NACT at the Second Affiliated Hospital of Wenzhou Medical University between January 2007 and August 2014. Clinicopathological variables were recorded, including age at diagnosis, stage, tumor grade and size. Details of treatment was documented for retrospective analysis. Clinicopathologic data was detailed in Table 1. None of these patients had received chemotherapy, immunotherapy, hormonal therapy or radiotherapy before the specimen collection. Paired tumor samples from each patient were obtained during cervical biopsy (pre-chemotherapy) or surgery (post-chemotherapy). Hematoxylin and eosin-stained slides of the cervical tumor from each patient were reviewed for confirmation of pathological features, as well as to select suitable tissue blocks for immunohistochemistry analysis.

**Table 1:** Clinical characteristics of cervical squamous cancer patients

Parameter	Responders (*n* = 17)	Non-responders (*n* = 18)	*P*
Median age (years)	47 ± 8	44 ± 9	NS*
FIGO stage			
IB2	8 (IB2)	11(IB2)	NS*
IIA2	9 (IIA2)	7 (IIA2)	
Differentiation			
G1	6	7	NS*
G2	10	10	
G3	1	1	
Primary tumor size (cm)	4.53 ± 1.24	4.17 ± 1.41	NS*

*P > 0.05, Comparison between the responder and non-responder cohorts

### NACT and therapeutic effect evaluation

All eligible patients received one or two courses of cisplatin-based NACT, as previous described [19]: cisplatin, 60 mg/m^2^ on day 1; 5-fluorouracil, 750 mg/m^2^ on day 1; mitomycin (8 mg/m^2^) on day 1 for one or two courses, every 28 days. All of these chemotherapeutics were administrated via uterine artery injection. The chemotherapy response was evaluated by measuring the tumor’s two dimensions (the longest diameter and its perpendicular diameter) with magnetic resonance imaging or other radiographic means (CT scan and ultrasound). Eighteen cases (51.4%) were measured by MRI, 4 cases (11.4%) were measured by CT scan and 13 cases (37.2%) were measured by ultrasound. The chemotherapy response was determined two weeks after completion of neoadjuvant chemotherapy, according to the WHO criteria [20]. Complete response (CR) was defined as the complete remission of the tumor. Partial response (PR) was defined as least a 50% decrease in the tumor volume. Stable disease (SD) meant a steady state or a response less than 50%, and progressive disease (PD) was defined as an unequivocal increase of at least 25% in the tumor volume. The patients with CR or PR were defined as chemotherapy responders, while the patients with SD or PD were deemed as chemotherapy non-responders. We evaluated the response after the first cycle chemotherapy, if the result showed response, we then did the second one. Totally, there are 18 cases under-going one cycle and 17 cases under-going two cycles. All patients were treated with radial hysterectomy and bilateral pelvic lymphadenectomy two to three weeks after completion of the NACT regimen as described previously [19].

### Western blotting

Samples were homogenized and lysed in Laemmli buffer with a cocktail of protease inhibitors. The total protein concentrations were quantified by the bicinchoninic acid protein assay (Thermo Scientific, Rockford, IL). Equal amounts of total protein were resolved by sodium dodecyl sulfate- polyacrylamide gel electrophoresis, transferred to a nitrocellulose membrane under constant voltage and blocked with tris buffered saline with tween (TBST) containing 5% non-fat dried milk. Primary antibodies and secondary antibodies were diluted in TBST and applied with a washing step in between. Proteins were detected using the Amersham ECL western blotting detection kit (GE Healthcare, Piscataway, NJ). Primary antibodies used including: anti-Trx 1 (CST, USA; 1:1000), anti-Prx 1(Abcam, USA; 1:2000), anti- Prx 2 (Abcam, USA; 1:1000), anti- β-actin (Abcam, USA; 1:5000). ImageJ software was used to quantify western blot bands in this study. ImageJ is a Java-based image analysis package widely used in quantitating visual results such as bands on gels or photomicrographs.

### Immunohistochemistry

Formalin-fixed paraffin embedded tissues were cut into sections of 4 μm and then mounted onto poly-L-lysine-coated slides. In briefly, after de-paraffinized in xylene and rehydrated through graded ethanol solutions, sections were boiled in a 10 μmol/L citric buffer suolution (pH 6.0) in a microwave oven for 10 min, followed by incubation with 3% hydrogen peroxide in methanol to suppress the endogenous peroxidase activity and overnight incubation with primary antibodies (Trx1, CST, USA; 1:200; Prx1, Abcam, USA; 1:200, Prx 2, Abcam, USA; 1:200) at 4 °C. Subsequently, the sections were incubated with pre-diluted secondary antibody (Santa Cruz, USA) for 2 h at room temperature, followed by further incubation with 3,3-diaminobenzidine tetrahydrochloride. Finally, the slides were counterstained with hematoxylin. Appropriate positive and negative controls were stained in parallel. For negative controls, primary antibodies were replaced with phosphate-buffered saline solution. Human cervical cancer tissues were used as a positive control.

### Evaluation of immunoreactivity

Trx 1, Prx 1 and Prx2 protein were detected primarily in cytoplasm and partially in cytomembrane. Assessment of expression of Trx1, Prx-1 and Prx2 staining was determined as previously described [19]. In brief, expression of the these markers was determined by an individual labeling score considering percent and staining intensity of positive cells by two independent assessors blinded to the study end points. [21] Intensity of stained cells was graded semi-quantitatively into four levels as following: 0 (no staining); 1 point (weak staining:  light yellow); 2 points (moderate staining: yellow brown) and 3 points (strong staining: brown). The percentage was scored as following: 0 (0 to 5%), 1 point (6 to 24%), 2 points (25 to 49%), 3 points (50 to 74%), and 4 points (75 to 100%). Intensity and fraction of positive cell scores were multiplied for each marker and thus got the immunoreactive score.

### Statistical analysis

The Kolmogorov-Smirnov test of normality was applied. Continuous variables were presented as mean ± standard deviations and non-normally distributed variables were presented as median (P25- P75). Since the patient profile between the NACT responsive and non-responsive group displayed a normal distribution, a Student t-test (age and tumor size) or chi-square test (FIGO stage and Histological grade) was used for analysis. Protein expression between the two groups displayed a non-normal distribution and was evaluated by non-parametric tests such as the Wilcoxon test. The software of SPSS 16.0 (SPSS Inc., IL) was used for statistical analysis. A *P-*value less than 0.05 were considered statistically significant.

## Results

### Patient characteristics

The first cohort consisted 13 bulky squamous cervical cancer patients. The median age was 49y (range 36~74y), and stage IB1 and stage IIA1 was 76.9 and 23.1%, respectively.

The second cohort consisted 35 patients with bulky stage IB2~IIA2 squamous cervical cancer treated with NACT. The median age was 44y (range 25~63y). Nineteen tumors were stage IB2 (54.3%), and sixteen cases were stage IIA2 (45.7%). Of the 35 patients identified, 5 patients (14.3%) showed a complete response and 12 patients (34.3%) showed a partial response, while 13 patients (37.1%) had a stable disease and other five patients (14.3%) showed progressive disease. Thus, 17 cases were chemo-sensitive (48.6%) and 18 were chemo-resistant (51.4%). There were no significant differences in patients or tumor characteristics between the responder and non-responder cohorts (Table 1). Clinicopathologic characteristics are shown in Table 1.

### Trx 1, Prx1 and Prx2 protein were over-expressed in squamous cervical cancer

**Fig. 1:**
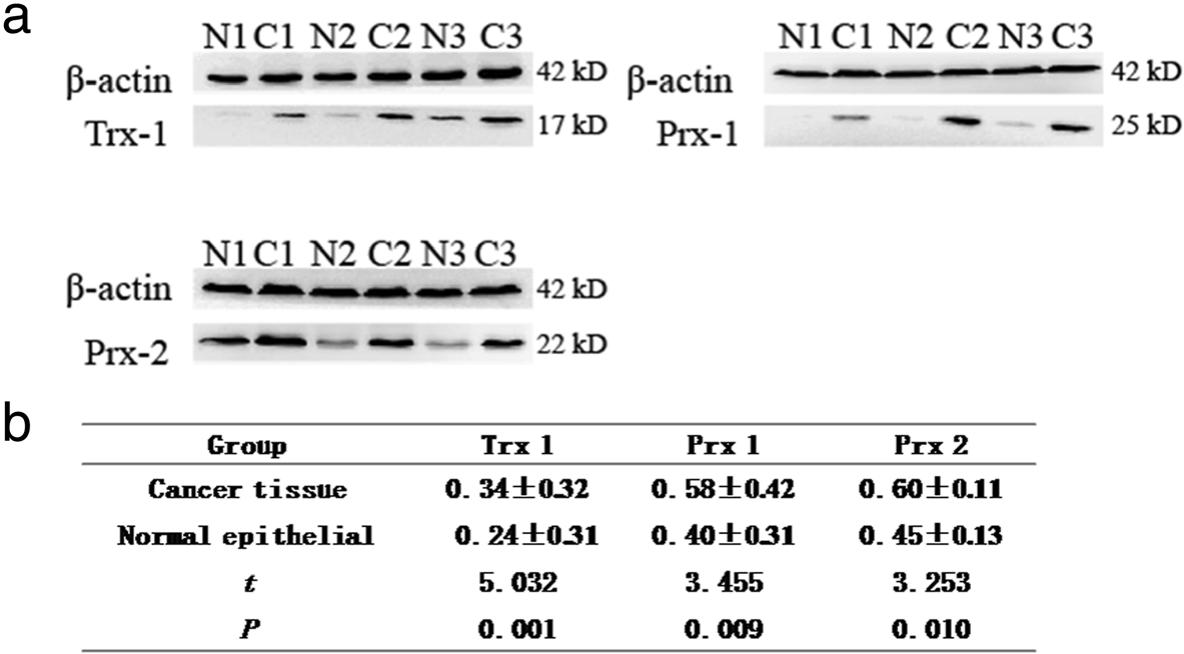
Western blot analysis of Trx 1, Prx 1 and Prx 2 expression in cervical squamous cancer and adjacent normal tissue. (**a**, **b**) Trx1, Prx 1 and Prx 2 protein expression were markedly up-regulated in squamous cervical cancer tissues compared to adjacent normal tissues. Actin protein level was used to validate equal sample loading

**Fig. 2:**
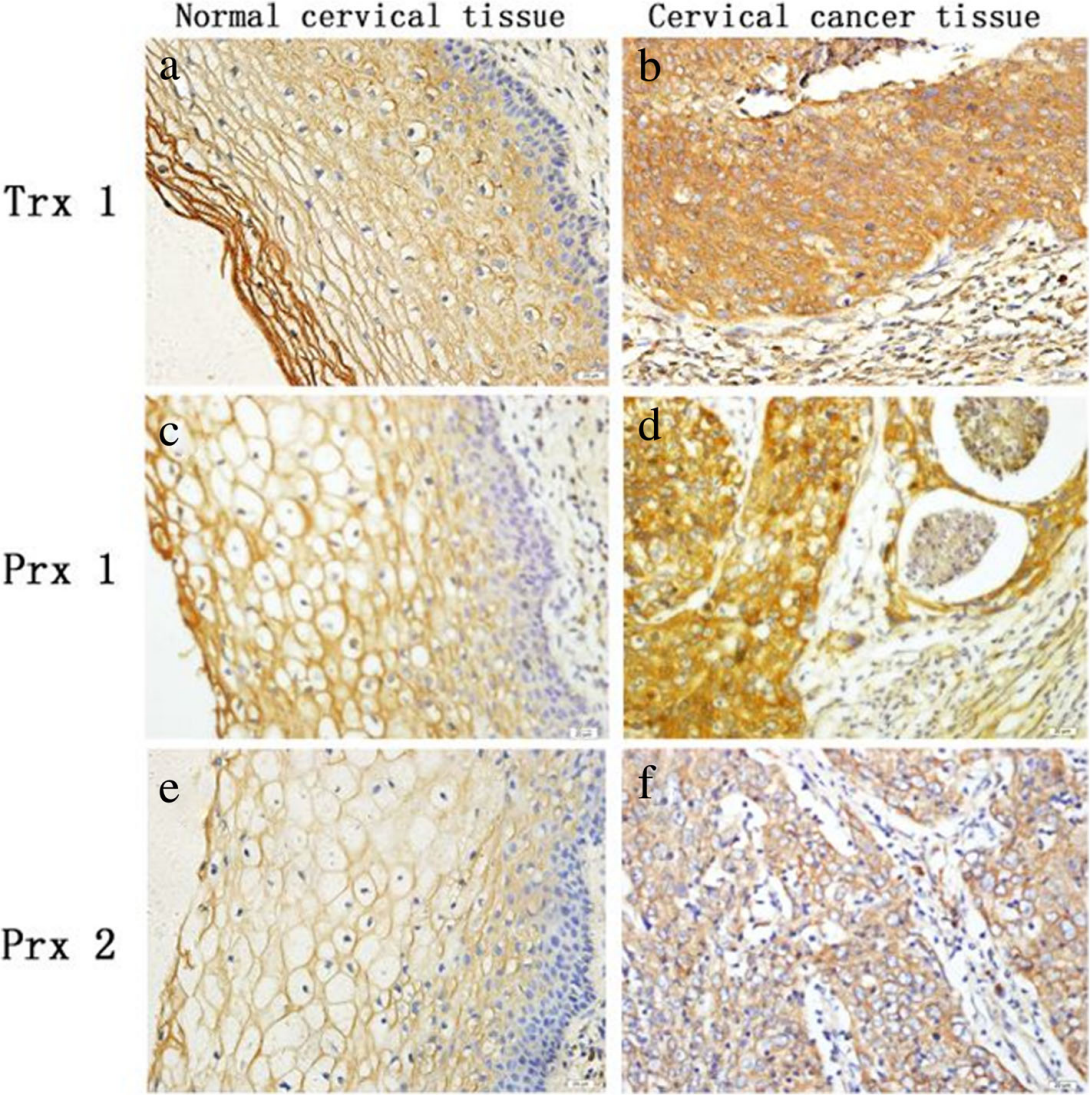
The expression of Trx 1, Prx 1 and Prx 2 proteins in cervical squamous cancer squamous cervical cancer and adjacent normal tissue by immunohistochemistry analysis (SP staining, × 400). Trx 1 (**a**, **b**), Prx 1 (**c**, **d**) and Prx 2 (**e**, **f**) proteins were detected primarily in cytoplasm and partially in cytomembrane. The expression of Trx 1, Prx 1 and Prx 2 was much stronger in cervical squamous cancer compared to basal layer cells of adjacent normal tissues

Initially, the expression of Trx 1, Prx 1 and Prx 2 in 13 cases of squamous cervical cancer and adjacent normal tissue was investigated by Western blotting. As shown in Fig. 1, all of these three indicated proteins were over-expressed in squamous cervical cancer tissues compared to adjacent normal tissues. The expression and localization of Trx 1, Prx 1 and Prx 2 in squamous cervical cancer and adjacent normal tissue were further examined by immunohistochemistry. A strong cytoplasmic or membranous expression of Trx 1 was observed in all 13 squamous cervical cancer samples analyzed, and generally limited to tumor cells (Fig. 2). The expression pattern of Prx 1 and Prx 2 in squamous cervical cancers was similar to Trx 1. Interestingly, with regard to adjacent normal tissue, moderate staining of Trx 1 was observed in superficial layer cells and intermediate layer cells of cervical epithelial among all the 13 samples, while only three cases showed positive staining in basal layer cells, in which two cases showed weak staining and the third case showed moderate staining. Similarly, all of the adjacent normal tissues (13/13, 100%) showed weak or moderate staining for Prx 1 and Prx 2 in superficial layer and intermediate layer of cervix epithelia, only 3 cases (23.1%) expressed weak or moderate staining in basal layer of cervix. Since, cervical epithelial cells in basal cells may be the source of malignant transformed cells, we compared the different expression of these three proteins between cervical cancers samples and the basal layer cells of adjacent normal tissues. Quantification of Trx 1, Prx 1 and Prx 2 staining showed that Trx 1, Prx 1 and Prx 2 immunoreactive scores were much higher in squamous cervical cancer compared to basal layer cells of adjacent normal tissues (*P* = 0.007, *P* = 0.01, *P* = 0.01, respectively), (Table 2). Figure 2 illustrates the immunostaining for all markers studied in squamous cervical cancer tissues and adjacent normal tissues.

**Table 2:** The expression of Trx 1, Prx 1 and Prx 2 proteins in tumor cells of cervical cancer and the basal layer cells of adjacent normal tissues

Group	n	Trx 1	Prx 1	Prx 2
Cancer cell	13	10.5(8.00–12.00)	7.0(4.00–8.00)	10.5(8.00–12.00)
Normal basal cell	13	0.0(0.00–3.25)	0.0(0.00–3.50)	2.0(0.00–6.00)
Z		−2.694	−2.578	− 2.561
*P*		0.007	0.01	0.01

Data are expressed as median (P25-P75)

* *P* < 0.05

### Trx 1, Prx 1 and Prx 2 protein were upregulated after NACT

**Fig. 3:**
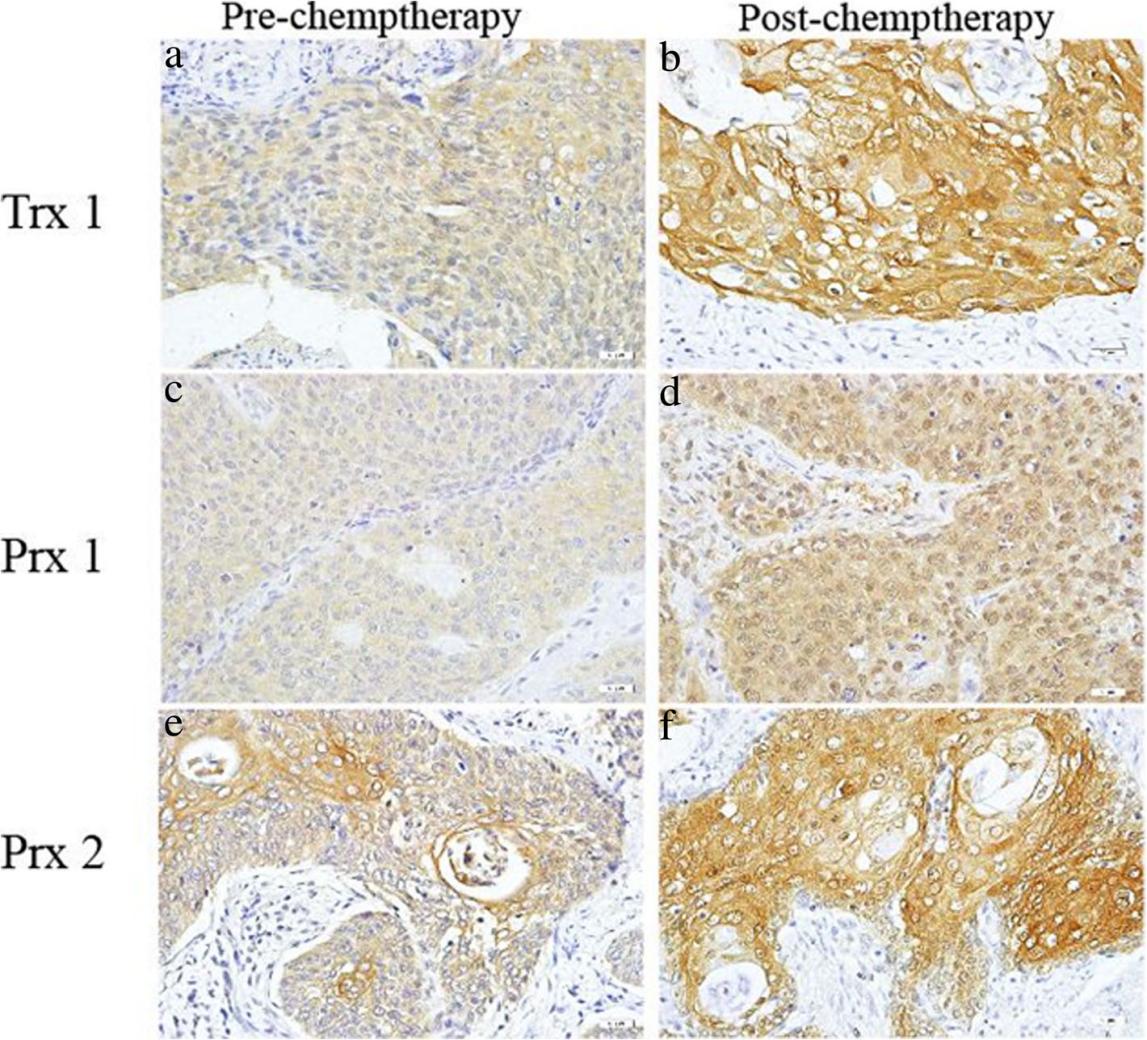
The expression of Trx 1, Prx 1 and Prx 2 in paired samples of cervical squamous cancer as compared to corresponding pre- chemotherapy and post- chemotherapy by immunohistochemistry analysis (SP staining, × 400). The expression of Trx 1, Prx 1 and Prx 2 was significantly up-regulated in post-chemotherapy tissues (**b**, **d**, **f**) compared to pre-chemotherapy tissues (**a**, **c**, **e**)

To investigate the effect of chemotherapy on the expression of Trx 1, Prx 1 and Prx 2 in squamous cervical cancer samples, the expression of Trx 1, Prx 1 and Prx 2 was performed in 35 matched primary squamous cervical cancer biopsies before and after cisplatin-based NACT using immunohistochemistry. Pre-chemotherapy cervical cancer tissues consistently showed weak or moderate positive staining of Trx 1, Prx 1 and Prx 2, while post-chemotherapy tissue consistently showed moderate or intense positive staining (Fig. 3). Using a Wilcoxon test, the expression of Trx 1, Prx 1 and Prx 2 were significantly stronger in post-chemotherapy tissues compared to pre-chemotherapy tissues, (*P* = 0.000, *P* = 0.001, *P* = 0.010, respectively) (Table 3), suggesting Trx 1, Prx 1 and Prx 2 protein were implicated in squamous cervical tumor responses to chemotherapy.

**Table 3:** The expression of Trx 1, Prx 1 and Prx 2 in cervical cancer cells pre- and post- chemotherapy

	n	Trx 1	Prx 1	Prx 2
Pre-chemotherapy	35	6.0(4.00–8.00)	6.0(4.00–8.00)	8.0(5.50–8.25)
Post-chemotherapy	35	9.0(8.00–12.00)	8.0(8.00–12.00)	12.0(8.00–12.00)
Z		−3.973	− 3.215	−3.313
*P*		0.000*	0.001*	0.010*

Data are expressed as median (P25-P75)

* *P* < 0.05

### Increased expression of Trx 1, Prx1 and Prx2 was associated with chemoresistance

To explore the role of Trx 1, Prx 1 and Prx 2 in the development of chemo-resistance, the expression of Trx 1, Prx 1 and Prx 2 in pre-chemotherapy cervical cancer tissues between chemotherapy responders and non-responders was examined by immunohistochemistry. As shown in Table 4, the expression of Trx 1, Prx 1 and Prx 2 in pre-chemotherapy cervical cancer tissues was markedly stronger in chemotherapy non-responders than that in responders, suggesting patients with high levels of Trx 1, Prx1 and Prx2 were more resistant to cisplatin-based NACT than those with low protein expression (*P* < 0.05, respectively).

**Table 4:** The expression of Trx 1, Prx 1 and Prx 2 in cancer cells of pre-chemotherapy cervical cancer between chemotherapy-response and non-response group

Group	n	Trx 1	Prx 1	Prx 2
Response	17	4.0(4.00–8.00)	6.0(4.00–8.00)	8.0(4.00–8.00)
Non-response	18	8.0(4.50–8.00)	8.0(8.00–12.00)	8.0(6.50–11.25)
Z		−2.199	−2.364	−2.024
*P*		0.028*	0.018*	0.043*

Data are expressed as median (P25-P75)

*P < 0.05

### The correlation between Trx 1 and Prx1/2 in squamous cervical cancer

The correlation between Trx1 and Prx1/2 in 13 cervical squamous cancer samples without chemotherapy and 35 pre-chemotherapy cervical squamous cancer samples were further explored. Interestingly, our results showed that there was a positive correlation between Trx 1 and Prx 2, but not between Trx 1 and Prx 1, suggesting Trx 1 and Prx 2 may function together in cervix carcinogenesis and chemoresistance (Table 5).

**Table 5:** The relevance between Trx 1 and Prx 1/2 in tumor cells of cervical squamous cancer

Group	n	Trx 1	
		r	*P*
Prx 1	48	0.04	0.79
Prx 2	48	0.31	0.04*

*P < 0.05

## Discussion

Aberrant reactive oxygen species levels has been well document to the initiation and progression of cancer. Numerous types of cancer cells are characterized by an increase in reactive oxygen species production, and often exhibit an altered redox environment compared with normal cells [7, 16]. Trx 1 is essential for the protection against oxidative stress, for the maintaining the balance of the cellular redox status and for the regulation of differentiation and cell fate [22]. This wide range of cellular functions leads into its involvement in a variety of diseases, including cancer. In fact, an increased level of Trx 1 is found in many primary human cancers, which may contribute to the development of tumor progression [11]. Trx 1 can stimulate tumor cell proliferation, inhibit apoptosis, promote tumor growth and decrease cancer patient survival [10]. Consistent with its functional role in other cancers, in the current study, we detected, for the first time, an increased expression of Trx 1 in squamous cervical caners, suggesting Trx 1 may play an important role in the development of cervical cancer.

Trx 1 exerts most of its antioxidant properties through peroxiredoxins [22]. Trx 1 reduces the oxidized form of Prxs, and reduced Prx in turn scavenges ROS, such as H_2_O_2_. Similar to Txr 1, increased levels of Prxs have been demonstrated in many human cancers, including cervical cancer. Prx 1 has been reported to exert its tumor- promoting function in numerous types of human cancer, and appears to be mediated through its interaction with several cancer-associated signal pathways, such as increasing the expression of vascular endothelial growth factor and activating c-Jun and AP-1, inhibiting epithelial-mesenchymal transition through the inhibition of E-cadherin and suppressing apoptosis through the inhibition of apoptosis signal-regulating kinase 1 activity [16]. Increased expression levels of Prx 2 have been found in breast prostate cancer, in prostate, and in esophageal cancer [16]. With respect to cervical cancer, Prx 2 protein levels were found to be progressively up-regulated from normal tissue to cervical intraepithelial neoplasia (CIN1, CIN2, and CIN3), and cervical cancer [23, 24]. In agreement with previous literature, we found that Prx 1 and Prx 2 protein were over-expressed in squamous cervical caners compared with basal layer of normal cervical tissues, suggesting a possible role for Prx 1/2 as a tumor promoter in cervical cancer. Collectively, these results supported the notion that cervical cancer cells, compared with normal cells, had higher reactive oxygen species levels as by-products of their metabolism. To survive from this redox status, the levels of thioredoxin peroxidase system, including Trx 1, Prx 1 and Prx 2, were up-regulated. The mechanism by which this system exerts its oncogenic action in cervical cancer is still poorly understood, and further studies are required.

As mentioned above, cervical squamous cancer cells may display variable profiles with respect to their redox-generating and -adaptive systems, and this unique capability of cancer cells may contributed to the development of resistance to chemotherapy, as these treatments are strongly dependent on ROS-induced cytotoxicity [4]. Recently, Trx 1 was widely documented contribution to the development to chemo-resistance. Trx 1 was found to be up-regulated in paclitaxel-resistant ovarian cancer A2780 cells and bortezomib-resistant multiple myeloma cells compared with their parental cells. [12, 25]. Inhibition of Trx 1 by small interfering RNA or a Trx 1 inhibitor can markedly sensitize diffuse Large B cell lymphomas cells to doxorubicin-induced cell growth inhibition [26] and enhance the growth inhibitory actions of cisplatin in pancreatic ductal adenocarcinoma [13]. Trx 1 promoted paclitaxel-induced drug resistance by increasing FOXO1 transcriptional activity and suppressing drug-induced apoptosis in ovarian cancer A2780 cell [12]. Additionally, Trx is downstream of Smad7 in a pathway that confers a growth advantage to pancreatic cancer cells and that increases their resistance to cisplatin-mediated apoptosis [13]. Our current study found that Trx 1 was markedly up-regulated after chemotherapy, suggesting Trx 1 involved in chemotherapy response in cervical squamous cancer. Moreover, patients with high levels of Trx 1 were more resistant to cisplatin-based NACT than those with low protein expression, which supported the recently highlighted roles of Trx 1 in the development of chemoresistance.

In addition to Trx 1, an increasing body of evidence demonstrates that elevated levels of Prx 1 and Prx 2 are often associated with chemoresistance to various drugs. High levels of Prx 1 and Prx 2 correlated with cisplatin chemoresistance in gastric cancer SNU638 cells [27], in human erythroleukemia K652, in human breast cancer MCF-7 cells [28], and in human ovarian carcinoma SKOV-3 cells [28], as increased levels of this antioxidant inhibited apoptosis. Elevated Prx 1 induced resistance to docetaxel treatment through suppression of FOXO1-induced apoptosis in lung cancer A549 xenograft tumors [29]. Furthermore, in gastric carcinoma cells, Prx 2-specific antisense vectors restored the induction of pro-apoptotic pathways following cisplatin treatment, confirming the important role of Prx 2 in the resistance process [30]. Our previous study detected up-regulation of Prx 1 in squamous cervical cancer tissue after NACT relative to the level before chemotherapy by using proteomics profiling [15]. In the current study, our data provided evidence that high expression of Prx 1 and Prx 2 was significantly correlated with a poor response to chemotherapy in squamous cervical cancer. These findings re-confirm our notion that thioredoxin peroxidase system confers the development of drug resistance. Chemo-resistant cervical cancer has higher levels of Trx 1 and Prxs, which hamper the therapeutic efficacy by scavenging the ROS involved in the mechanism of chemotherapeutic drugs.

As with any retrospective study, these are some limitation in this study. At first, response was not analyzed pathologically, which providing more information on the remaining cells and stroma regarding the expression of the proteins of interest. Secondly, the chemotherapy response was evaluated by three radiographic means (magnetic resonance imaging, CT scan and ultrasound), which may cause heterogeneity. In addition, this retrospective study, because of its nature that may have not well assessed the response and the different number of chemo cycles, suggest that could be an association between higher basal or prechemotherapy expression of these proteins with either the response or the number of chemo cycles.

## Conclusions

In conclusion, our current study detected an increased level of Trx 1, Prx 1 and Prx 2 in cervical squamous cancer, suggesting a possible role for thioredoxin peroxidase system as a tumor promoter in cervical squamous cancer. Furthermore, our results demonstrated that the higher expression of Trx 1, Prx 1 and Prx 2 in pre-chemotherapy could predict a poor response to NACT in cervical squamous cancer. Measuring Trx 1, Prx 1 and Prx 2 in biopsy samples before treatment may contribute to more effective management of bulky cervical cancer. More studies with large samples are needed to determine the predictive role of Trx 1, Prx 1 and Prx 2 in cervical cancer chemotherapy.

## Data Availability

The datasets used and/or analyzed during the current study available from the corresponding author on reasonable request.

## Abbreviations

CIN: Cervical intraepithelial neoplasia
CR: Complete response
FIGO: International Federation of Gynecology and Obstetrics
NACT: Neoadjuvant chemotherapy
PD: Progressive disease
PR: Partial response
Prxs: Peroxiredoxins
ROS: Reactive oxygen species
SD: Stable disease
Trx 1: Thioredoxin 1
